# 我如何治疗异基因造血干细胞移植后晚发重症肺炎

**DOI:** 10.3760/cma.j.issn.0253-2727.2023.09.004

**Published:** 2023-09

**Authors:** 于谦 孙, 晓军 黄

**Affiliations:** 北京大学人民医院，北京大学血液病研究所，国家血液系统疾病临床医学研究中心 Peking University People's Hospital & Peking University Institute of Hematology, National Clinical Research Center for Hematologic Disease, Beijing 100044, China

异基因造血干细胞移植（allo-HSCT）是治疗血液系统疾病的有效手段甚至是唯一治愈手段。肺部感染是移植后主要合并症，发生率高达40％～70％[1]–[2]。而一旦发生肺炎，相较于免疫功能正常人群而言，allo-HSCT患者更容易发展为重症肺炎[3]–[4]。

造血干细胞移植后晚发重症肺炎（late-onset severe pneumonia, LOSP）是指在造血干细胞移植后晚期（>3个月）发生的重症肺炎，通常迅速进展至呼吸衰竭，病死率高达60％～70％，严重影响造血干细胞移植患者的生存[5]–[9]。目前对于LOSP的病因、流行病学、病理生理机制等还缺乏深入的认识，因而在LOSP的诊断、治疗、预防等策略上仍存在许多挑战。在本文中，我们依据北京大学血液病研究所的临床工作经验并参考文献资料，对造血干细胞移植后LOSP的诊治策略进行介绍。

一、典型病例

患者男性，因慢性粒-单核细胞白血病（WHO分型CMML-1，FAB分型CMML-MP，CPSS评分高危），于2019年9月行弟供兄全相合造血干细胞移植（血型B+供B+），移植后18 d中性粒细胞植活，移植后32 d血小板植活；移植后60 d发生急性移植物抗宿主病（Ⅳ度，皮肤、肠道），经糖皮质激素及巴利昔单抗治疗后达到完全缓解。

患者于移植后112 d出现咳嗽、咯白痰等症状，血常规示WBC 1.93×10^9^/L、HGB 53 g/L、PLT 21×10^9^/L，C-反应蛋白（CRP）160.05 mg/L。口服莫西沙星400 mg/d，症状无改善，于移植后119 d（起病后7 d）收住入院。入院时动脉血气氧分压（PaO2）91 mmHg；咽拭子PCR检测提示呼吸道合胞病毒（RSV）、副流感病毒弱阳性；胸部高分辨CT提示右肺中叶支气管壁增厚毛糙，周围肺组织可见多发模糊斑片影及磨玻璃影；双肺散在模糊结节影及斑片影（图1A）。予哌拉西林他唑巴坦、静脉注射免疫球蛋白（IVIg）、伏立康唑治疗，并继续予以复方磺胺甲恶唑预防肺孢子菌肺炎。移植后121 d（起病后9 d）患者开始出现高热，亚胺培南/西司他丁钠、利奈唑胺、替加环素等广谱抗生素治疗无效，CRP持续>100 mg/L，于移植后124 d（起病后12 d）进展至1型呼吸衰竭，诊断为“异基因造血干细胞移植后晚发重症肺炎”，予IVIg、更昔洛韦等治疗，但患者仍持续发热、呼吸衰竭无改善；移植后131 d（起病后19 d）胸部CT示双肺多发磨玻璃影、实变等病变加重（图1B），予以地塞米松7.5 mg/d抑制炎症因子风暴，当天体温降至正常，次日低氧血症改善、CRP降至正常；移植后138 d（起病后26 d，地塞米松治疗第8 d）复查CT示肺炎较前明显好转（图1C）。地塞米松逐渐减量过程中病情无反复，顺利出院。移植后187 d（起病后75 d，地塞米松治疗57 d）门诊随访，无症状，CT示肺部炎症明显吸收（图1D）。

二、allo-HSCT后LOSP的定义及临床特点

（一）定义

发生于造血干细胞移植后晚期（3个月后）的重症肺炎我们称其为移植后LOSP，参照中华医学会呼吸病学分会制订发布的《中国成人社区获得性肺炎诊断和治疗指南（2016年版）》[10]进行诊断。与非移植人群发生的重症肺炎相比，allo-HSCT后LOSP具有一些独特的特征：①死亡率极高：美国Mayo诊所的一项研究显示，移植后合并急性呼吸窘迫综合征（ARDS）患者的28 d死亡率为46.6％[11]；本中心的系列报道显示LOSP的死亡率55％～58％[5]–[6]；②allo-HSCT后LOSP可能具有与其他重症肺炎不同的病原谱：allo-HSCT后早期（3个月内）的肺炎可由细菌、真菌、病毒各种病原导致，其中病毒常以巨细胞病毒（CMV）、EB病毒（EBV）等疱疹类病毒为主；而移植后晚期重症肺炎可能主要与呼吸道病毒（流感病毒、呼吸道合胞病毒等）感染有关 [1]。基于以上特殊的临床特点，allo-HSCT后LOSP概念的单独提出有助于临床医生重视此类并发症，具有重要的实践意义。

**图1:**
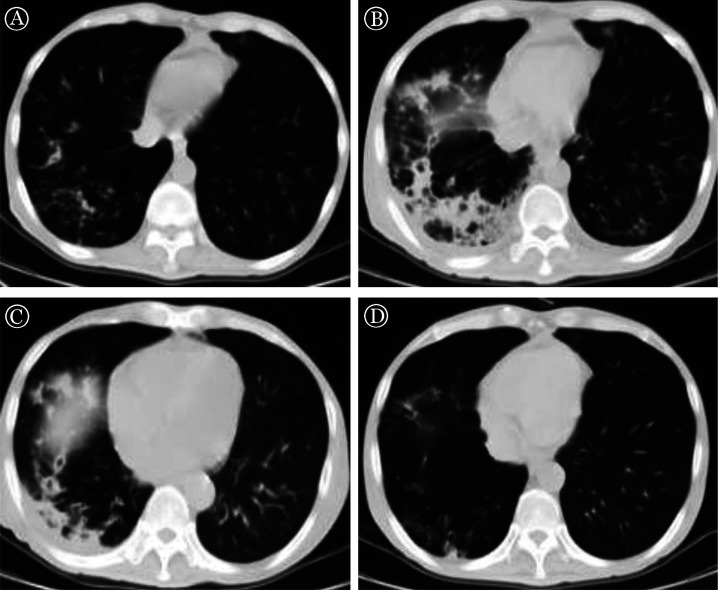
一例异基因造血干细胞移植后晚发重症肺炎患者治疗前及治疗后肺部高分辨CT图像 A：起病7 d；B：起病19 d，诊断1型呼吸衰竭；C：起病26 d，地塞米松治疗1周，呼吸衰竭好转；D：起病75 d，无症状，肺部炎症明显吸收

（二）临床特点

文献报道造血干细胞移植后肺炎的发生率为40％～70％[1]–[3]，但缺乏重症肺炎的发病率数据。美国Mayo诊所的一项研究显示，造血干细胞移植后ARDS的总体发生率为5％，自体造血干细胞移植（auto-HSCT）患者为2.7％，allo-HSCT患者为15.6％[11]。本中心的系列回顾性分析提示，LOSP的发生率为1.3％～2.4％[5],[7]。需要注意的是，由于对allo-HSCT后LOSP的研究较少，LOSP的实际发生率缺乏准确的流行病学数据。

allo-HSCT后LOSP具有以下一些特点：①发生在移植后相对晚期，本中心数据显示中位发生时间为移植后213～218 d[5],[7]。②以进行性呼吸困难为初发症状，低氧血症迅速进展，病程通常2～3周内快速进展。③胸部影像学检查常表现为双侧渗出性改变。④病原检出率低：国外一项前瞻性多中心的研究总结了移植后13个月内肺炎的病因，其中有近一半患者肺炎病因不明[1]，这与我们的数据较一致，即使行支气管镜、支气管肺泡灌洗等检查，病原检测阳性率也较低[7]–[9]。⑤检出病原以病毒为主：2021年本中心报道100例造血干细胞移植后重症肺炎患者，55例（55.0％）呼吸道病原体检测阳性，病毒为最常见的病原体（27例，49.1％），RSV是造血干细胞移植后重症肺炎最常见的病因（占29.6％）[9]，提示LOSP的病原较为复杂，呼吸道病毒可能是其重要病原。⑥缺乏特异性治疗手段，病死率高：经验性或针对检出病原的特异性抗感染治疗效果不佳，病情进展快，病死率较高，生存率仅为31％～45％[5]–[9]。

三、对allo-HSCT后LOSP的认识

（一）重症肺炎的发生

allo-HSCT后LOSP的发生、发展机制并未完全阐明，由于患者多为抗细菌/真菌治疗无效，故LOSP很可能由病毒或非感染性因素引起。北京大学血液病研究所的系列研究显示病毒感染可能是主要病因：2016年报道的50例造血干细胞移植后LOSP患者的血液/肺泡灌洗液样本中，28例（56％）病原体检测阳性，其中19例（68％）为病毒[8]；2017年更新报道的68例造血干细胞移植后LOSP患者，32例（47.1％）有病原学证据（血培养、痰培养及肺泡灌洗液培养），其中17例（53％）为病毒[7]；2018年报道77例造血干细胞移植后诊断肺部感染患者的病原体分布情况，其中最常见的感染诊断是病毒性肺炎（54/77，70％），RSV 19例（35％）[12]；2021年更新报道100例造血干细胞移植后LOSP患者，55例（55％）检出呼吸道病原体，其中病毒最常见（27例，49.1％），RSV是造血干细胞移植后重症肺炎最常见（占29.6％）的病因之一[9]。提示在LOSP患者中应重视呼吸道病毒感染的可能，如RSV、流感病毒、副流感病毒、偏肺病毒等，显著不同于移植后早期常见的CMV/EBV等疱疹病毒。

值得注意的是，无论是国外研究还是本中心研究，约一半的移植后肺炎患者未能发现病原体。病毒是HSCT后重症肺炎的主要检出病原；不过，病毒阳性与病毒阴性患者在临床特点及预后方面极为相似，这提示：①病毒可能不是真正导致重症肺炎的病因；②病毒感染伴有其他的病理生理因素加重肺炎进展；③由于缺乏特异性药物，因此病毒所致重症肺炎与其他病因导致的重症肺炎在临床上无法区别；④最近新型冠状病毒的流行导致约10.9％移植后患者发生重症肺炎，其临床特点、影像学特征与移植后重症肺炎较为相似（未发表数据），但抗病毒治疗疗效较好，间接提示肺炎是病毒引起。

（二）重症肺炎的病理生理

我们对LOSP的病理生理机制还知之甚少。既往研究提示，感染导致肺损伤主要包括以下两方面机制：①病原体直接接触肺泡上皮、肺泡内皮细胞造成肺损伤；②病原体诱导的宿主免疫反应相关损伤。

近年来越来越多的证据提示病原体诱导的宿主免疫反应相关病理肺损伤参与了重症肺炎的发生：①新型冠状病毒重症肺炎患者体内肺部免疫细胞（CD4^+^T细胞、单核细胞）过度活化，产生大量炎症因子，通过正反馈循环的机制形成细胞因子风暴（CRS），由CRS导致的多器官功能衰竭是重症新冠肺炎患者死亡的重要原因[13]；②在甲型H1N1流感病毒小鼠肺损伤模型中，多种细胞因子（IL-6、TNF-α、CXCL10）水平显著升高，同时单核细胞来源的肺巨噬细胞数量显著升高[14]。我们在allo-HSCT后LOSP患者中，也发现了炎症因子（尤其是IL-6）显著升高，且炎症因子的水平与LOSP的严重程度及预后相关（尚未发表）；③适时应用糖皮质激素可使部分重症肺炎患者获得显著改善[6]–[7]，提示炎症反应在重症肺炎发生发展中可能起到重要作用。鉴于当前抗病毒药物及治疗手段的缺乏，基于炎症反应的治疗策略有可能有一定作用。

四、我如何治疗allo-HSCT后LOSP

（一）重视造血干细胞移植后肺炎的进展

对于移植后晚期患者，一旦出现上呼吸道感染症状，应警惕其进展为肺炎甚至LOSP的可能。对此类患者，在治疗上呼吸感染的过程中，应予以密切监测。尤应重视病原学检测，尤其是呼吸道病毒的检测。由于病原学检出率较低，应反复多次进行病原学检测。

（二）支持治疗

由于病情进展迅速、快速获得病原学结果困难，造血干细胞移植后发生LOSP患者早期启动经验性抗微生物治疗十分必要。对于病原不明确者，经验性抗感染策略常需覆盖细菌、肺孢子菌、流感病毒，而在新型冠状病毒流行之后，甚至可能需要覆盖新型冠状病毒。对于已明确病原者，及时采取针对性的治疗，如针对流感病毒、新型冠状病毒的治疗。此外，由于多数患者缺乏特异性治疗手段，支持治疗尤其是呼吸支持治疗在重症肺炎的管理中具有重要地位。

（三）抗炎治疗

鉴于炎症反应在LOSP的病理生理过程中起到重要作用，抗炎治疗可能是LOSP的潜在策略之一。糖皮质激素具有显著的抗炎作用，可以在炎症反应的早期减轻渗出、毛细血管扩张、白细胞浸润和炎症因子释放，在后期可抑制毛细血管和纤维母细胞的增生、减轻肺渗出及肺纤维化，改善患者通气功能、缩短住院时间。例如，在重症社区获得性肺炎、重症流感病毒肺炎、SARS、新型冠状病毒肺炎等疾病群体中均有糖皮质激素治疗改善生存的报道[15]–[17]。然而，糖皮质激素在发挥抗炎、免疫抑制作用的同时，不利于病原的清除，甚至增加二重感染的风险，也有糖皮质激素增加重症病毒性肺炎患者死亡率的报道[18]–[19]。因此糖皮质激素的剂量及使用时机相当具有挑战性。

本中心前期回顾性研究提示，糖皮质激素的使用时机和剂量与LOSP患者的预后相关，肺炎发生晚期（≥1周）小剂量甲泼尼松龙（≤2 mg·kg^−1^·d^−1^）与较好的预后相关[7]。近期本中心的回顾性分析显示，抗病毒治疗疗程与糖皮质激素使用的时机与LOSP的预后相关，早期开始抗病毒（启动时间<10 d）且糖皮质激素使用较晚（距离发病>10 d）的患者60 d生存率为91.4％，而晚期开始抗病毒治疗且早期使用糖皮质激素的患者仅为21.4％[6]。

因此，糖皮质激素应尽可能延后使用，在无特异性抗病毒治疗的条件下，应尽可能在诊断2周后应用；若有明确病原（如新型冠状病毒），则建议病毒转阴2次以上应用。糖皮质激素剂量不宜也无需太大，建议甲泼尼龙1 mg·kg^−1^·d^−1^即可，若有效通常2～3 d可见需氧量显著改善。

综上，基于目前对LOSP病原、发病机制方面的理解，我们推荐临床实践中采用抗微生物+抗炎治疗的综合策略治疗allo-HSCT后LOSP（表1）。

**表1:** 异基因造血干细胞移植后晚发重症肺炎（LOSP）的综合治疗策略建议

早期识别
对移植后晚期呼吸道感染患者警惕进展为LOSP可能
综合治疗
①反复多次进行病原检测，重视呼吸道病毒检测；
②病情评估及监测：充分结合临床症状、体征、炎症指标、影像学变化；
③治疗：抗微生物治疗+支持治疗为基础，覆盖可能病原（病原明确者，针对性抗感染治疗；病原不明确者，经验性感染策略应覆盖细菌、肺孢子菌、流感病毒）
酌情使用糖皮质激素抗炎治疗
①无明确病原的患者，应尽可能在诊断2周后使用糖皮质激素；
②有明确病原（如新型冠状病毒）的患者，建议病毒转阴2次以上应用糖皮质激素；
③糖皮质激素剂量建议甲泼尼龙1 mg·kg^−1^·d^−1^

五、展望及未来研究方向

我们推测allo-HSCT后LOSP是一组以呼吸道病毒为主要病原导致的急性发作、急进性进展至呼吸衰竭、伴有高死亡率的疾病。然而，allo-HSCT后LOSP还有很多问题尚待解决：①探寻早期、准确鉴定LOSP病原的新方法；②深入研究重症肺炎的病理生理机制，探寻新的生物标记物以指导或实现精准糖皮质激素使用或其他病理生理治疗；③探讨LOSP的预防策略（病毒疫苗或促进免疫重建等）。

总之，allo-HSCT后LOSP的流行病学、快速诊断手段、预后评估指标、特异性治疗策略等方面依然还需要更多更深入的研究。而上述问题的研究将有助于进一步理解重症肺炎的病理生理过程并有助于优化LOSP的防治策略。
